# The Shape of the Urine Stream — From Biophysics to Diagnostics

**DOI:** 10.1371/journal.pone.0047133

**Published:** 2012-10-16

**Authors:** Andrew P. S. Wheeler, Samir Morad, Noor Buchholz, Martin M. Knight

**Affiliations:** 1 Engineering and the Environment, University of Southampton, Southampton, United Kingdom; 2 School of Engineering and Materials Science, Queen Mary University of London, London, United Kingdom; 3 Department of Urology, Newham University Hospital, Plaistow, London, United Kingdom; University of Arizona, United States of America

## Abstract

We develop a new computational model of capillary-waves in free-jet flows, and apply this to the problem of urological diagnosis in this first ever study of the biophysics behind the characteristic shape of the urine stream as it exits the urethral meatus. The computational fluid dynamics model is used to determine the shape of a liquid jet issuing from a non-axisymmetric orifice as it deforms under the action of surface tension. The computational results are verified with experimental modelling of the urine stream. We find that the shape of the stream can be used as an indicator of both the flow rate and orifice geometry. We performed volunteer trials which showed these fundamental correlations are also observed in vivo for male healthy volunteers and patients undergoing treatment for low flow rate. For healthy volunteers, self estimation of the flow shape provided an accurate estimation of peak flow rate (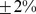). However for the patients, the relationship between shape and flow rate suggested poor meatal opening during voiding. The results show that self measurement of the shape of the urine stream can be a useful diagnostic tool for medical practitioners since it provides a non-invasive method of measuring urine flow rate and urethral dilation.

## Introduction

The analysis of urine flow rate is important in the diagnosis of urinary conditions ranging from neuromuscular pathologies to bladder outlet obstruction [1]–[3]. This has led to the development of a variety of uroflowmetry techniques including gravimetric methods, orifice outflow devices such as the Uflow and more sophisticated rotating disc flow meters [4]–[8]. It is therefore surprising that there are no published studies in the field of urology, which describe the characteristic shape or wave pattern made by the urine stream as it exits the urethral meatus and whether this shape contains potential diagnostic information. In this study we show how the shape of the urine stream, which arises due to surface-tension driven capillary-waves that initiate at the meatal opening, contains useful diagnostic information about the urine flow rate and meatal dilation.

Liquid jet flows have received significant attention in the published literature, and there has been a substantial amount of work which has elucidated the surface-tension driven effects which develop in these flows (a thorough review can be found in [9]). Short wavelength capillary instabilities can often lead to break-up of a liquid column [10]–[12]. Whilst this may be observed further down in the urine stream, the wavelengths at the start of the stream tend to be much longer than the cross-sectional size of the meatus and thus the urine stream is initially relatively stable. In the present study we develop computational fluid dynamics modelling to explain for the first time, the characteristic wavelike shape of the urine stream and its relationship to flow rate and the size and shape of the urethral meatus. In so doing we identify two novel diagnostic parameters which can be derived from simple non-invasive visual inspection of the flow stream. These parameters are then examined with both healthy volunteers and a clinically relevant patient cohort.

## Computational Methods

For the computational simulations of the urine stream we make use of a novel method which replicates the jet shape from an orifice by simulating the unsteady development of a 2-dimensional droplet deforming under the action of surface tension. This model is described next.

### Modelling non-axisymmetric jets

**Figure 1:**
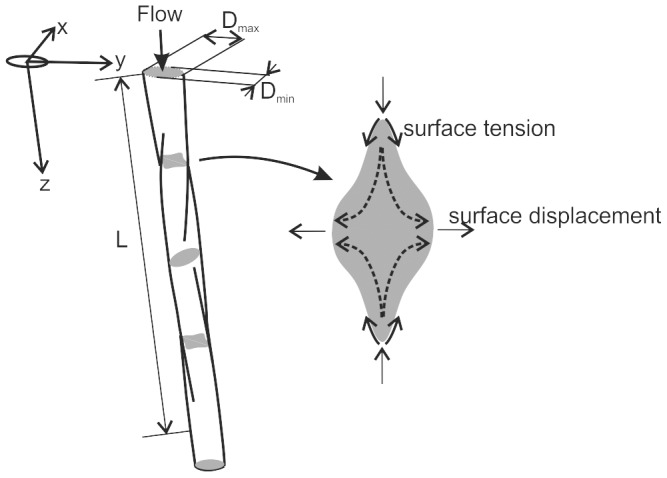
Sketch of the liquid jet flow exiting an elliptical orifice.

When a liquid jet issues from a non-cylindrical aperture, the jet formed tends to undergo large deformations under the action of surface tension. For instance, for a jet issuing from an elliptic aperture, the flow pattern is similar to that shown in Fig. 1. The inital shape of the jet will closely match the aperture shape, and the surface tension will act to reduce the local surface curvature, thus accelerating the flow radially inwards in regions of high convex curvature. However, in order to conserve mass flux, the flow must also accelerate radially outwards elsewhere on the jet surface. Thus, along the axis of the jet, the jet surface forms a wave-like pattern with displacements in orthogonal directions (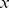, 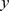). The wavelength (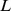) of these oscillations is dependent on the jet flow rate, aperture geometry and surface tension (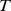). As the jet develops downstream, the jet surface oscillates under the action of surface tension, and the opposing action of the radial and tangential momentum in the jet. For a viscous fluid, the viscosity damps out these oscillations, so that at distances far downstream the jet effectively becomes cylindrical. On the other hand, if viscous effects are small, and the forces due to surface tension are large in comparison to the momentum in the jet, then the jet surface can become unstable and break-up due to the amplification of capillary waves.

Consider the jet flow in Fig. 1, where the minimum dimension of the jet at the exit of the aperture is 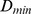. For the cases described here, we find that the initial wavelength (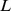) to be around thirty times larger than 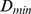. The pressure differences due to surface tension are inversely proportional to the radius of curvature of the jet surface both in the streamwise direction and in the x-y plane (see Fig. 1). Because L is over an order of magnitude greater than 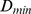, the radius of curvature in the streamwise direction will be much larger than the radius of curvature in the x-y plane (which will be approximately 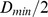), and its contribution to the internal pressure within the jet is neglected. Similarly, the gradients in pressure along the axis of the jet will be at least an order of magnitude smaller than pressure gradients within the x-y plane and so we neglect streamwise pressure gradients. Therefore the pressure within the jet is assumed to be solely due to the curvature of the surface in the x-y plane. We will also make the assumption that the streamwise 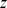-component of velocity (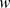) is a constant, and set by the cross-sectional area of the jet and the volume flow-rate. This assumption will generally hold if the streamwise pressure gradients are small, and the action of streamwise body forces (such as gravity) are also small. A further simpification will be to neglect the second derivatives of velocity in the streamwise direction, which is reasonable when 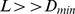 since in this case the jet surface will not deform rapidly along the jet axis. This latter assumption in effect neglects the shear forces due to velocity gradients in the streamwise direction. The streamwise flow velocity in the jet is typically around 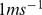, which gives a skin friction coefficient due to the action of aerodynamic drag on the fluid stream of around 0.012 (assuming a laminar air boundary-layer at a Reynolds number based on 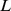 of 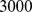). This gives a surface shear of around 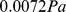, which can be compared to the pressure due to surface tension which will typically be around 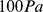. For this investigation the aerodynamic drag forces could reasonably be neglected as being several orders of magnitude less than the surface tension forces. Given these assumptions the steady-state incompressible Navier-Stokes equations at any streamwise plane along the jet axis become
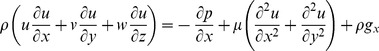
(1)

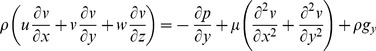
(2)

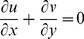
(3)Where 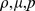 are the fluid density, viscosity and pressure, respectively, and 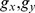 represent body forces (such as surface tension) acting on the fluid elements.

Now consider elements of fluid within a two-dimensional droplet which is deforming in time (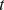) under the action of surface tension. The motion of these fluid elements is described by the unsteady incompressible Navier-Stokes equations in two dimensions, which are:
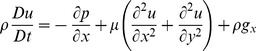
(4)

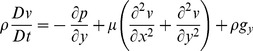
(5)

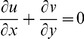
(6)One can immediately see that the deformation of the jet surface, in this simplified case, can be described by the deformation of a two-dimensional droplet which is deforming unsteadily in time under the action of surface tension. The streamwise 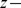
axis for the jet flow is simply the time (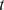) axis for the droplet, scaled by the streamwise jet velocity (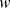):
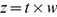
(7)


### Application to physiologically relevant flow

Making use of these assumptions, a computational method developed here is solved for the unsteady development of a 2-dimensional ‘droplet’, whose initial shape was determined from the orifice geometry. The computational solution algorithm comprised of a finite volume, 2nd order, pseudo-compressibility, dual-time stepping scheme with 2nd and 4th order smoothing. The effects of laminar viscosity were added by determining the strain field normal to the axis of the jet, and including the shear forces in the finite volume formulation. The Reynolds number based on wavelength 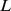 was typically around 4000. A correction for gravitational effects was also added, by scaling the droplet area to account for a fixed volumetric flow rate at each streamwise plane of the jet. Both the laminar viscosity and gravitational effects were found to make little difference to the solutions. The liquid properties used were those of water (surface tension = 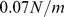, viscosity = 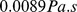). The surface tension forces were applied using body forces around the edge of the droplet. At the end of each physical time-step, the edge of the droplet was displaced according to the calculated velocity, and a new computational grid was fitted to the new shape.

**Figure 2:**
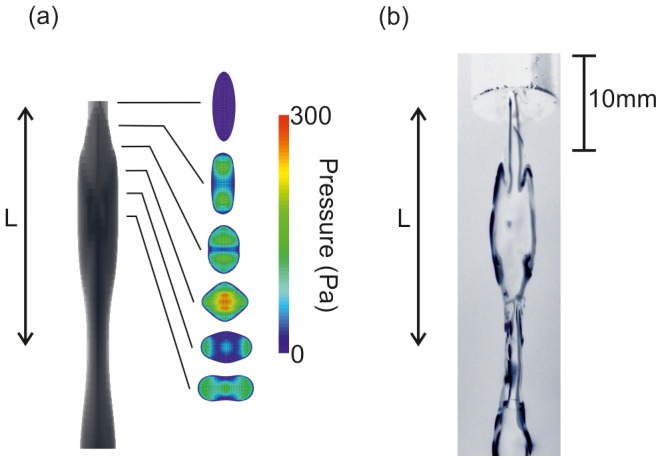
Experimental and computational models describe the shape of the urine flow pattern. (a) Computational fluid dynamics (CFD) indicates the pressure distribution and shape of a liquid stream exiting from an elliptical orifice such as the urethral meatus. (b) Experimental models with a rigid walled elliptical orifice produced the characteristic flow pattern of the urine stream (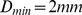, 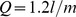).

**Figure 3:**
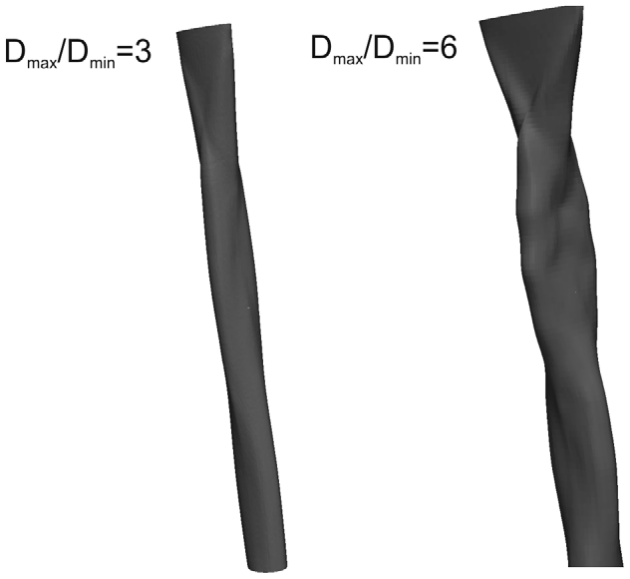
Computational modelling solves the 3-dimensional jet flow from a wide range of orifice shapes. Two examples are shown here for aspect ratios (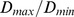) of 3 and 6, and 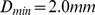, 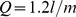.

The resulting simulated flow patterns match closely with those generated experimentally for water exiting an identical elliptical orifice at physiologically relevant flow rates (Fig. 2). The computational and experimental models both produce flow patterns which show a characteristic initial wavelength (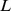) representing the distance between the orifice and the first pinch point as shown in Figure 2. Further examples of computed jet flows are shown in Fig. 3.

**Figure 4:**
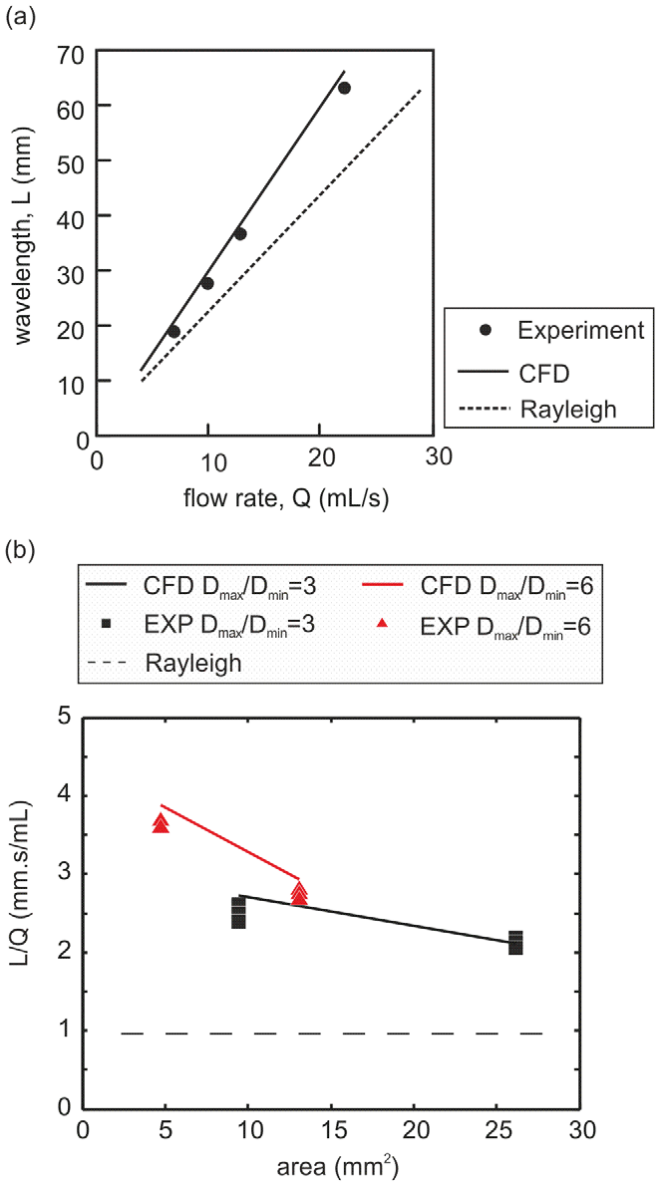
Experimental and computational models show the influence of flow rate and orifice size and geometry on the shape of the jet flow. (a) For any given orifice, there was a perfect linear correlation between flow rate, 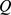 and wavelength, 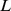, with excellent agreement between the experimental and CFD analysis as shown for an orifice with cross sectional area and 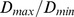 of 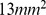 and 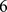 respectively. The relationship between flow rate and wavelength was also estimated using the method of Rayleigh which assumes small amplitude perturbations from a cylindrical jet in contrast to the current CFD analysis. For large amplitude oscillations which occur when aspect ratios 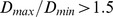, the Rayleigh method significantly under estimated the wavelength. (b) The dilation parameter, 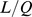, is dependent on the cross sectional area and the aspect ratio (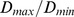) and therefore may be used to describe urethral/meatal opening. Comparisons between the CFD and experimental data for a range of orifice shapes show an excellent agreement in the values of 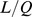.

Both the experimental and computational modeling show a very similar positive linear relationship between flow rate (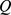) and wavelength (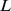) for a given orifice (Fig. 4). It was Lord Rayleigh [13] who first suggested that the characteristic wavelength (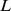) of a non-cylindrical liquid stream was directly proportional to the flow rate (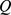) for small amplitude capillary waves, although this has never been discussed with relation to urine flow. Here we show that the method of Rayleigh, significantly underestimates the actual wavelength in this simulation of the urine flow pattern (Fig. 4(a)). This deviation from Rayleigh is because the assumption of small amplitude capillary waves is not valid when the orifice aspect ratios are substantially larger than unity, as in the case of the urethral meatus.

The computational and experimental modeling highlight the dependence of wavelength on orifice aspect-ratio ratio (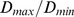) and cross sectional area (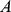). Thus variation in orifice size or urethral dilation between individuals or during the course of a single void will influence the flow pattern and the wavelength. Considering the analogy with the 2-d droplet oscillating with frequency 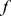, and traveling with a velocity 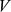, we see that the wavelength is given by Equation 8.
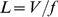
(8)
Equation 8 follows from Equation 7, where the unsteady development of a two-dimensional droplet describes the three-dimensional shape of the jet, and thus the wavelength L scales directly with the flow velocity V. This can also be expressed in terms of flow rate 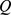, and orifice cross sectional area 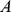, as in Equation 9.
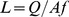
(9)Since, for a given liquid density, the oscillation frequency is solely a function of droplet shape, it follows that the ratio of wavelength to flow-rate (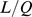) is purely dependent on the orifice area and aspect ratio (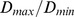) (the action of viscosity was found to be small in this case and does not influence the oscillation frequency). We therefore propose a new parameter, 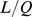, which is a measure of the meatal dilation or opening as shown in Fig. 4(b). Comparisons between the CFD and experimental data for a range of orifice shapes show an excellent agreement in the values of dilation parameter 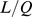 (see Fig. 4(b)), with values ranging from 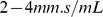. We will see later, from the in-vivo trials similar values for dilation parameter.

The computational and experimental modelling has therefore shown us that we would expect the urine stream wavelength 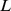 to be directly proportional to the flow-rate 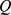 for a fixed meatal geometry and that the ratio 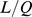 can be used as a measure of meatal dilation.

## Results and Discussion

### In-vivo trials

**Figure 5:**
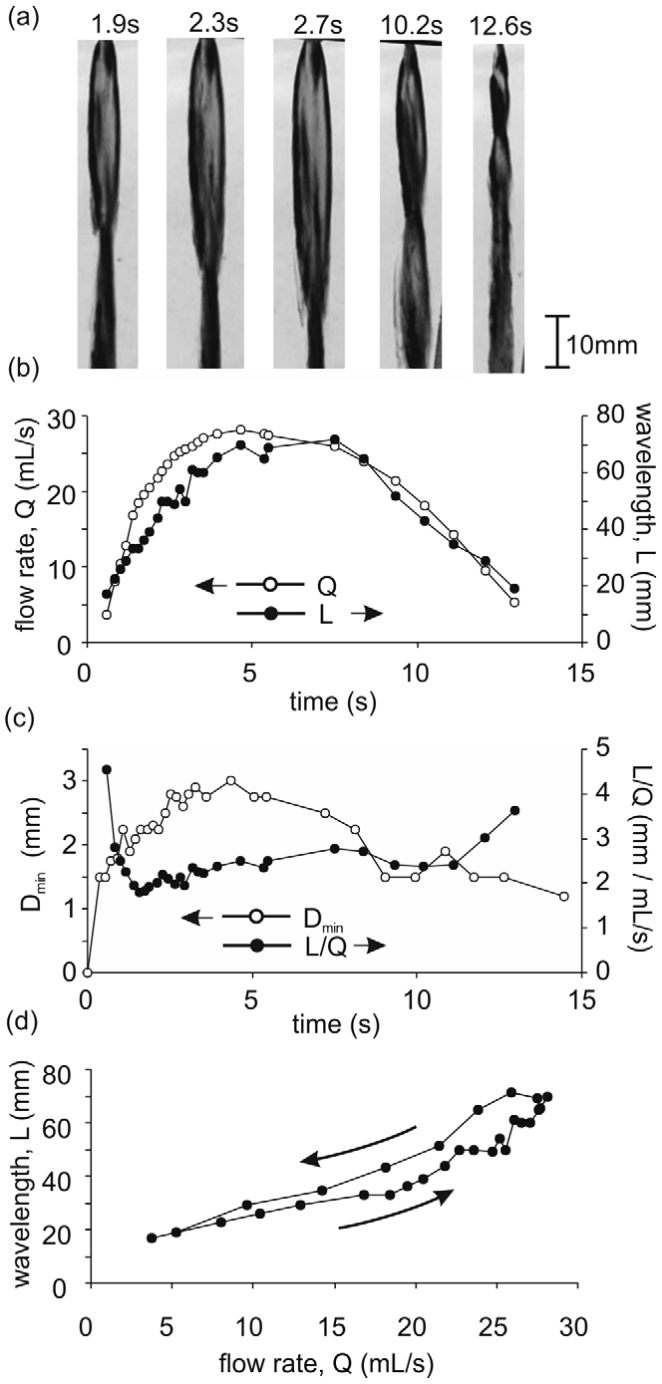
The human urine flow stream shows a characteristic pattern that is dependent on flow rate and orifice dilation as predicted by the experimental and computational modelling. (a) Selected video images from which the wavelength was measured by calibrating against a rule held alongside the flow stream. (b) Representative plot for an individual void showing the temporal change in instantaneous flow rate, 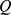, and wavelength, 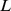. The wavelength is a function of both the flow rate and the shape and size of the meatus. (c) The opening of the urethral orifice during voiding was quantified by the minimum diameter of the meatal ellipse (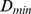) measured from the video images. The size and shape of the orifice is also characterised by the dilation parameter 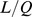, such that a reduction in 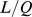 corresponds to an increase in opening. (d) The resulting plot of wavelength, 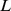, versus flow rate, 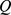, shows a clear correlation. Note that the relationship is non-linear due to the changes in the meatal opening.

Having developed the theory explaining the flow pattern associated with an elliptical orifice, we now examine the real flow pattern for urine exiting the meatus and the changes which occur during a single void. Figure 5(a) shows representative images taken from a video of a complete voiding event for a healthy male volunteer. The characteristic shape of the urine stream matches closely with that predicted by the experimental and computational models. Results show a typical temporal profile, such that the flow rate increases to a maximum value of 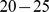 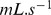 and then gradually reduces over the course of the void (Fig. 5(b)). This corresponds with a change in the wavelength which reaches a maximum of approximately 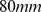 coinciding with the peak urine flow rate (Fig. 5(b)). There is a clear positive correlation between wavelength (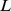) and flow rate (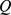) as predicted Fig. 5(d). The relationship is not purely linear, as illustrated by the change in 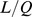 values during the course of the void (Fig. 5(c)). The 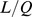 parameter reflects the orifice dilation, as quantified through the modelling Fig. 5(c), and thus the non-linearity indicates changes in the meatal dilation during the void. Indeed this was confirmed by measuring from the video images, the meatal opening in terms of the minimum diameter of the urine stream at the meatus (Fig. 5(c)). Thus during voiding the meatus opens under the flow pressure so that the aspect ratio reduces and cross sectional area increases, thereby influencing the wavelength (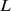) with an associated reduction in the dilation parameter (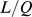). At the onset of voiding the pressure is sufficient to cause the meatus to rapidly open (Fig. 5(c)). However towards the end of the voiding the pressure drops gradually and the meatus slowly returns to its closed form due to the viscoelastic nature of the urethral tissue. This explains the difference between the descending and ascending curves in (Fig. 5(d)). Future studies may incorporate solid modelling of the urethral tissues to understand the temporal dynamics of meatal dilation during voiding and hence the effect on the flow pattern.

We then enlisted a group of 60 male patients who had been referred to a urologist due to low urine flow rate and suspected bladder outlet obstruction associated with prostatic enlargement. The patients were asked to record the maximum wavelength whilst voiding into a clinical urine flow meter. The flow meter used was a Smartflow (Albyn Medical). In addition, the same procedure was performed with a sample of 60 healthy male volunteers with no history of urinary flow problems. All participants from both groups reported the same characteristic urine flow pattern which evolved over the course of a single void in line with the flow rate, as shown in Fig. 5(b) 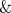 (c).

**Figure 6:**
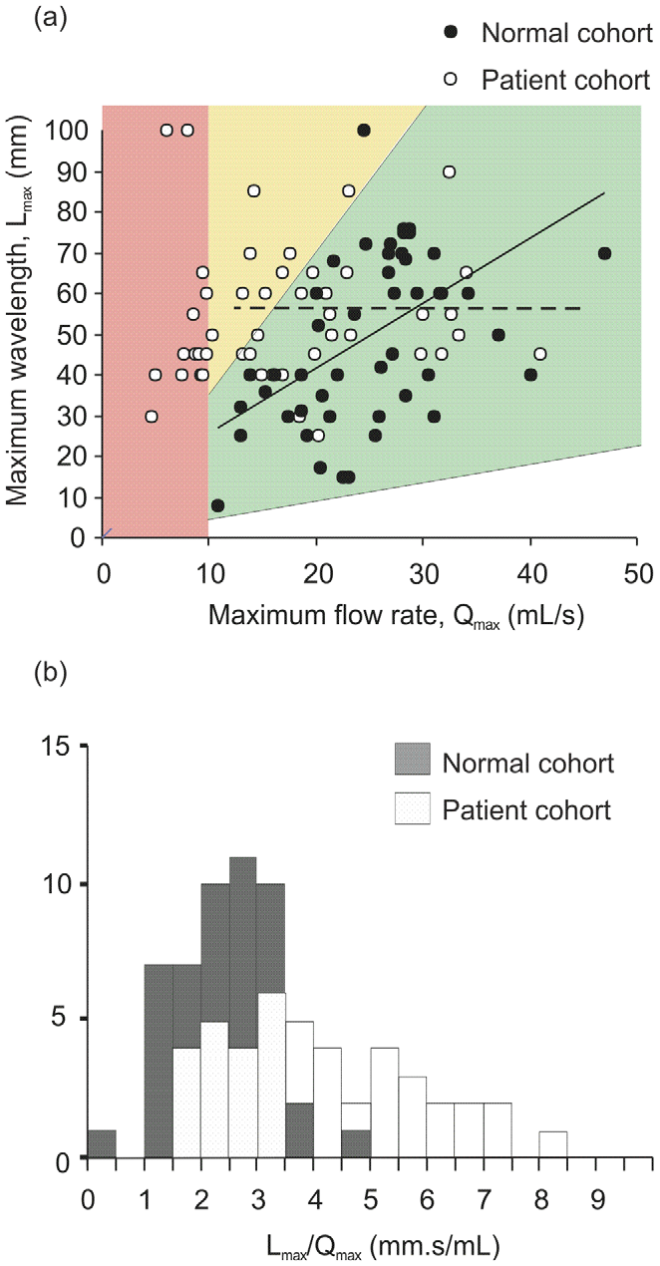
Self measurement of the maximum wavelength provides an estimate of the peak urine flow rate for healthy males, but requires individual calibration for patients undergoing treatment for urethral obstruction. (a) Plot of 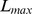 versus 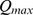 showing a statistically significant positive correlation for healthy men (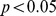, solid line) but not for the patient cohort (dashed line). A peak flow rate 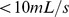 was considered as abnormally low (red region). The green region represents the 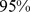 confidence envelope for 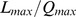 values based on data from the normal group. Individuals with 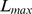 and 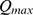 values within the yellow region have a normal flow rate but reduced urethral dilation. (b) Frequency distributions for the dilation parameter, 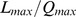, for the healthy and patient groups showing a statistically significant difference suggesting that the latter have reduced urethral opening.

For the group of healthy volunteers there was a statistically significant (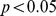) positive correlation (correlation coefficient 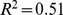) between peak flow rate (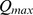) and maximum wavelength (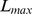)(Fig. 6(a)). The patient group showed no statistically significant correlation between 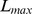 and 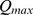 in contrast to the positive correlation for healthy volunteers (Fig. 6(a)). For the patient group, the dilation parameter (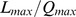) was statistically different (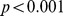) and exhibited greater variability than that for healthy men (Fig. 6(b)). Notably some patients with low peak flow rates showed higher values of the dilation parameter indicative of a reduced meatal opening. A reduction in a patient's meatal opening might be expected at very low flow rates where there is insufficient flow to fully open the meatus (Fig. 6, red region). Nonetheless, meatal dilation also appeared to be reduced in patients that have regained a more normal flow rate (Fig. 6, yellow region). It is possible that the reduced urethral opening reflects the greater average age of the patient cohort and associated age-related urethral stiffening [14]. However there was no correlation between age and dilation parameter with younger patients also showing high values. Thus an alternative explanation is that the chronic low flow rates in these patients may lead to urethra atrophy or constriction and that this persists even after the prostatic urethral obstruction causing the low flow rate is reduced. Although it is unclear for how long such an effect might persist, our data is supported by clinical experience which suggests that certain patients may benefit from surgical dilation of the urethra in order to regain a normal flow rate [15]. Thus, our data and the resulting nomogram shown in Fig. 6(a), helps to identify this subset of patients as those for whom 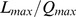 is greater than the 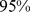 confidence limit of 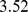, determined for healthy volunteers [16].

**Figure 7:**
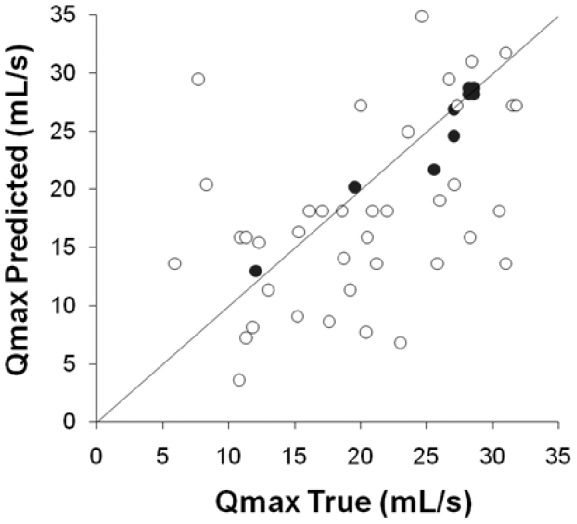
Flow rate measurement accuracy can be improved if an individual's dilation parameter is calibrated for. The open symbols show the predicted 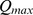 based on 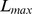 multiplied by the sample mean value of 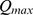/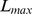. The closed symbols show the predicted 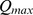 based on an individual's 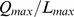 measured at the first recorded voiding event. The open symbols show the increased scatter if the sample mean 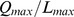 value is used rather than the individual's 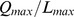.

Accurate estimation of an individual's peak urine flow rate based on measurements of maximum wavelength can be performed if an individual's meatal dilation is calibrated for. Self measurement of an individual's urine flow pattern and maximum wavelength can provide a simple non-invasive method for monitoring peak urine flow rate as part of the recommended practise of watchful waiting for patients with benign prostatic hyperplasia [17]–[20]. This has advantages over existing uroflowmetry techniques in that it is completely non-invasive, simple and cheap to implement and avoids inaccuracies associated with voiding in a clinical setting and obtaining data from a single void [7], [21]–[24]. For the group of healthy volunteers the statistically significant (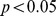) positive correlation gave the following relationship between 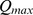 and 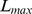


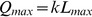
(10)where 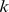 is found to be 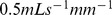. The accuracy of this estimate of 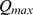 is 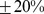 (2 standard deviations). However this can be greatly improved by precalibrating for an individual's meatal geometry. This could simply be achieved by requesting the patient to void into a standard urine flow meter to obtain the relationship between 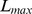 and 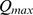. This approach would also provide a non-invasive measurement of an individual's meatal dilation during voiding. This is demonstrated in Fig. 7, which shows the predicted versus measured flow-rate for an individual, where the individual's dilation parameter has been calculated at one point, and used to predict the peak flow-rate at several other voiding events, using the individual's self measurement of 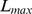. The figure shows the uncertainty in the estimate of 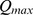 was improved to 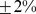 (2 standard deviations).

In this report we have applied an understanding of capillary wave phenomena in liquid jets to reveal the biophysics behind the characteristic shape of the urine flow stream and how this can be used as a simple non invasive means of measuring urethral opening and urine flow rate. The data obtained in the present study included inaccuracies caused by poor estimates of 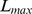 which are likely to be exacerbated by obesity, poor eye sight, or lack of manual dexterity. However despite the associated scatter there was still a statistically significant correlation between 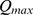 and 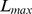 for healthy volunteers, showing that an individual's peak urine flow rate can be estimated from self measurement of maximum wavelength. Thus this technique can provide a simple non-invasive method for monitoring peak urine flow rate as part of the recommended practise of watchful waiting for patients with benign prostatic hyperplasia [17]–[20]

## Materials and Methods

### Ethics statement

All volunteers gave signed consent to the trial which was approved by the local ethics committee at Queen Mary University of London (Ethics Application No. QMREC2009/18). Healthy volunteers were recruited from staff and students within the University. Patients were assessed for urine flow rate as part of their standard on-going clinical treatment/monitoring for bladder outlet obstruction associated with benign prostatic enlargement as described in the text.

### In vitro Experimental Modelling

Rigid walled orifice geometries used in the experimental work were manufactured using a rapid-prototyping technique within a 0.1 mm tolerance. Orifice circumference was varied from 6 to 33 mm to reflect the physiological range in urethral dimensions. The nozzles were designed to have a length of 50 mm section to ensure a straight walled channel of fixed cross-sectional shape prior to the exit orifice, thus ensuring minimal expansion or contraction of the jet at the nozzle exit. Liquid water was supplied at mains pressure (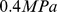) and preset physiological flow rates of 5 to 50 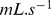.

### Imaging of the Male Urine Stream

Imaging of complete voiding events was conducted for a health male volunteer. A scale rule was held alongside and parallel to the urine stream to enable the instantaneous wavelength to be measured from the video images. The temporally varying flow rate was measured using a clinical gravimetric urine flow meter. Data was adjusted for the 0.2 second delay between the imaging of the wavelength at the meatus and urine flow rate being detected at the flow meter.

### Trials with Health Volunteers and Patients

Sixty healthy volunteers and sixty patients, mean ages 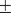
1 standard deviation were 26
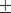
8 and 67
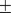
13 respectively, were asked to void normally into a clinical urine flow meter and to record the maximum wavelength in their flow stream. Individual data consisted of the maximum wavelength, the corresponding peak flow rate, the total voided volume and the subject's age. The patients,were at various stages of treatment with an alpha-1 receptor blocker (Tamsulosin) and/or an alpha-reductase inhibitor (Finasteride) to reduce the size of the prostate, such that some had regained a more normal peak flow rate (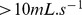) at the time of the trial. Procedures were conducted in private with approval of the local ethics committee.
